# Supplementary material for: Predicting visual function by interpreting a neuronal wiring diagram
Source: Nature. 2024 Oct 2;634(8032):113–23. doi: 10.1038/s41586-024-07953-5 (PMC11446822; doi:10.1038/s41586-024-07953-5)
Supplement: Supplementary file 6 — CRF and ERF predictions for individual TmY4 and TmY9 cells. Analogous to Supplementary Data 3, but for TmY target types. Shown are the top four monosynaptic pathways, the strongest pathway passing through each of the top ten intermediary types (ranking from Extended Data Fig. 7), and the trisynaptic pathway Tm1–TmY–Dm3–TmY (see the section entitled Prediction of spatial normalization). [file 41586_2024_7953_MOESM6_ESM.zip › DataS4/TmY9q/720575940619129502.pdf]

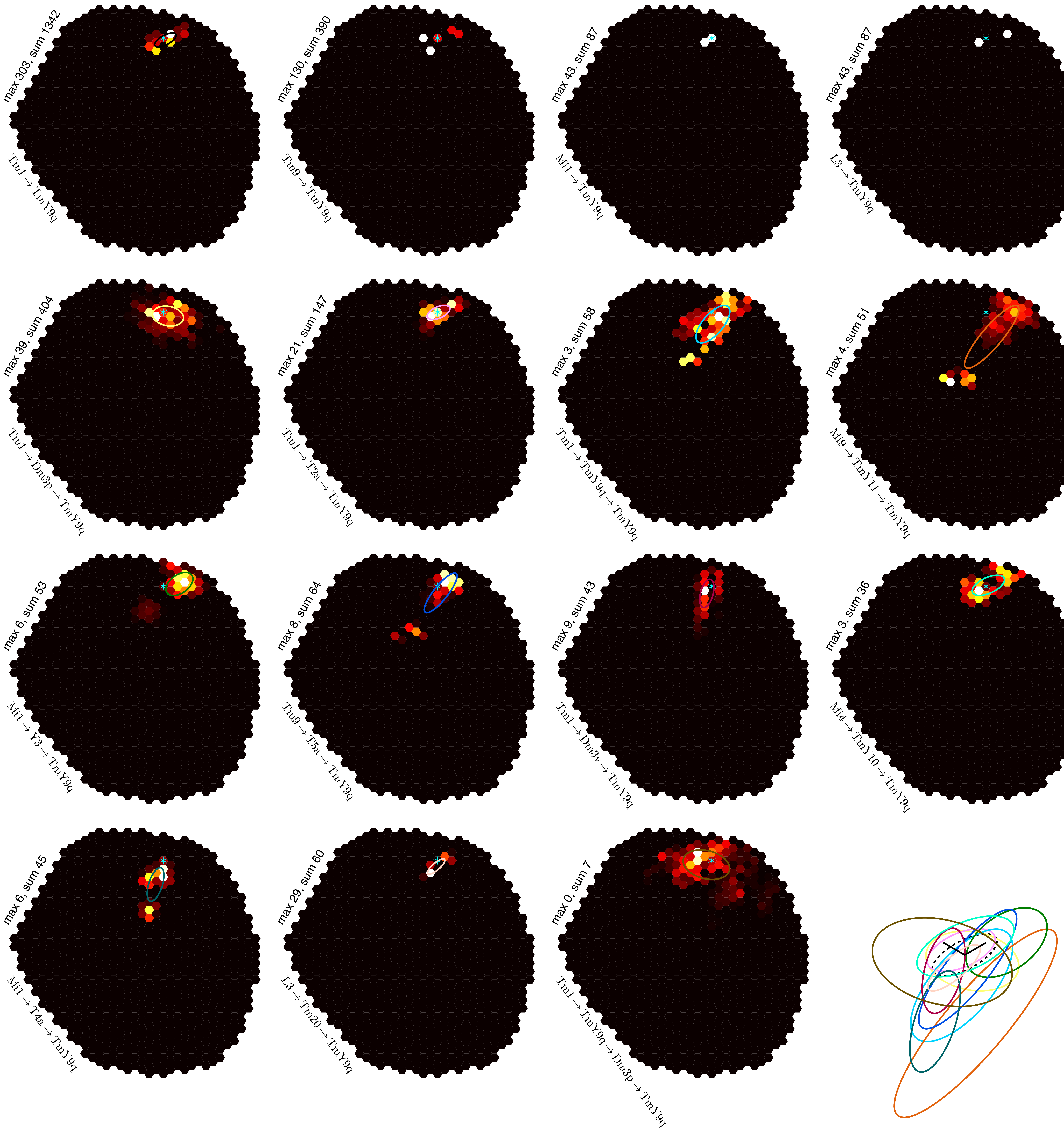

max 303, sum 1342
Tm1 → TmY9q
max 130, sum 390
Tm9 → TmY9q
max 43, sum 87
Mi1 → TmY9q
max 43, sum 87
L3 → TmY9q
max 39, sum 404
Tm1 → Dm3p → TmY9q
max 21, sum 147
Tm1 → T2a → TmY9q
max 3, sum 58
Tm1 → TmY9q → TmY9q
max 4, sum 51
Mi9 → TmY11 → TmY9q
max 6, sum 53
Mi1 → Y3 → TmY9q
max 8, sum 64
Tm9 → T5a → TmY9q
max 9, sum 43
Tm1 → Dm3v → TmY9q
max 3, sum 36
Mi4 → TmY10 → TmY9q
max 6, sum 45
Mi1 → T4a → TmY9q
max 29, sum 60
L3 → Tm20 → TmY9q
max 0, sum 7
Tm1 → TmY9q → Dm3p → TmY9q
